# Proportion of HIV exposed infants aged 0-6 months that missed nevirapine prophylaxis in Mulago National Referral Hospital, Uganda: a cross-sectional study

**DOI:** 10.1186/s12887-024-04600-w

**Published:** 2024-07-04

**Authors:** Nasambu Hellen, Rujumba Joseph, Mupere Ezekiel, Semitala Fred, Senyonga Ronald, Musoke Philippa

**Affiliations:** 1 Department of Paediatrics and Child Health, School of Medicine, College of Health Sciences, Makerere University, P. O. Box 7072, Kampala, Uganda; 2 Department of Internal Medicine, School of Medicine, College of Health Sciences, Makerere University, P. O. Box 7072, Kampala, Uganda

**Keywords:** HIV exposed, Infant, Nevirapine prophylaxis

## Abstract

**Background:**

Nevirapine prophylaxis has been found to lower the risk of HIV transmission in breastfed infants. While about 95% of HIV positive pregnant and lactating mothers use Antiretroviral therapy in Uganda, a smaller percentage of HIV exposed infants (HEI) receive nevirapine (NVP) prophylaxis. This study aimed to determine the proportion of HEI who missed NVP prophylaxis and associated factors.

**Methods:**

This was a cross-sectional study done using quantitative methods, conducted at Mulago National Referral Hospital (MNRH). A total of 228 mother-infant pairs were enrolled. The proportion of HEI who missed NVP, maternal, infant and health facility factors associated were determined using a pre-tested questionnaire. Bivariate analysis and binary logistic regression model were used to determine the proportion and factors associated with missing NVP prophylaxis.

**Results:**

The proportion of HEI who missed NVP prophylaxis was 50/228 (21.9%). Factors significantly associated with HEI missing NVP prophylaxis included delivery from outside government health facilities (AOR = 8.41; *P* = 0.001), mothers not undergoing PMTCT counselling (AOR = 12.01; P = 0.001), not on ART (AOR = 8.47; *P* = 0.003) and not having disclosed their HIV status to their partners (AOR = 2.80; *P* = 0.001). The HEI that missed nevirapine and were HIV positive were 35 (70.0%). The HEI that were HIV infected despite receiving nevirapine prophylaxis were 5 out of 40(12.5%).

**Conclusion:**

One in five HEI missed NVP prophylaxis and nearly three quarters of those who missed NVP prophylaxis were HIV infected. Improving uptake of nevirapine by HEI will require interventions that can aid to strengthen PMTCT counselling.

## Background

In Uganda, mother-to-child transmission of HIV accounts for up to 18% of all new infections and is the primary source of infections among children. In 2021 estimates indicate high rates of infection in children, with over 5955 children infected through vertical transmission [1]. For over two decades, the elimination of new HIV infections among children has been of paramount concern to the global HIV community [2]. Recommendations for reducing vertical transmission which accounts for the majority of pediatric infections in low resource settings were updated by WHO to include option B+. This requires all pregnant and breastfeeding HIV-infected women, regardless of CD4 cell count, to continue ART for life known as “option B+” while their infants receive daily NVP or zidovudine from birth to 4 to 6 weeks [3]. Both maternal ART and infant NVP prophylaxis strategies are safe and associated with very low breastfeeding HIV transmission [4]. However, the number of HIV Exposed Infants (HEI) who receive ART prophylaxis has stagnated at around 42% [5]. A recent study conducted at Mulago Hospital in 2019 studied factors influencing maternal adherence to infant’s NVP prophylaxis regimen [6] but did not establish the proportion of HEI that missed nevirapine prophylaxis and factors associated with missing NVP prophylaxis.

We determined the proportion of and factors associated with missing NVP prophylaxis within the first 72 hours of life among 228 HEI seen at Mulago Hospital, a national referral hospital in central Uganda.

## Methods

### Study design

The study data was part of a cross-sectional study done using mixed quantitative and qualitative methods of data collection conducted between April 2019 and December 2019. In this study, missing nevirapine (NVP) prophylaxis within the first 72 hours referred to not receiving NVP prophylaxis within the first 72 hours of life regardless of whether it is later received or not. For this publication, we focused on the quantitative data collected only.

### Study setting

The study was conducted at Mulago National Referral Hospital (MNRH), Kampala Uganda [in the immunization clinics and Acute Care Unit (ACU)]. Mulago Hospital is Uganda’s National Referral hospital and the teaching hospital for Makerere University College of Health Sciences. It is located in the central region and receives patients from all over the country. Kampala is the capital city of Uganda. The hospital is a government facility with a total bed capacity of 1500 in-patient beds, an inpatient turnover of 120,000 patients and attends to over 500,000 outpatients annually. Non-government facilities are privately owned. The Pediatric Department is made up of seven wards including ACU. The ACU runs an inpatient section and it admits a minimum of 500 infants and children annually. On average, about 30 HIV exposed infants are admitted per month. There’s a Provider initiated HIV testing and counseling (PIHTC) point where all mothers admitted at ACU have an opt out HIV test done after consent and pre-testing counselling is done. Lately, a DNA-PCR point of care machine has been introduced where HIV testing is done for children below 18 months of age and results are got immediately.

The immunisation clinic offers all recommended vaccines as per the Uganda National Expanded Program of Immunization (UNEPI) schedule. The clinic runs daily and an average of 70 infants and children under 5 years of age are immunized, of which, 10% are HIV exposed. The mother-infant pairs were recruited daily from Acute Care Unit and on all clinic days in the immunization clinics.

The study population included HIV sero-exposed infants aged 0–6 months and their mothers who were either attending the immunization clinic or were admitted due to various reasons at ACU in Mulago hospital during the study period. The study participants were HEI aged 0-6 months in Mulago hospital while the study respondents were their mothers.

The inclusion criteria were as follows:

1. HIV sero-exposed infants aged 0 to 6 months and their mothers who were either seen at the immunization clinic or were admitted at Acute Care Unit in Mulago Hospital during the study period regardless of whether they were registered in any PMTCT program or not.
2. Those whose mothers provided informed consent.

An infant who presented with a care taker who was not his/her mother if the mother was alive was excluded in the study.

### Study variables

The dependent variable was an HIV exposed infant who missed nevirapine prophylaxis.

The independent variables were factors associated with missing nevirapine prophylaxis and these are:

Infant factors: prematurity, low birth weight, infant hospitalization at birth.

Maternal factors: age, religion, education level, undergone PMTCT counselling, attended ANC, disclosure of HIV status to partner, mode of delivery, place of delivery, household income.

### Sample size estimation

#### Sample size estimation for proportion of HEI who missed nevirapine prophylaxis

A study done by Muhumuza et al. in 2013 analysed data abstracted from health facility records of pregnant or lactating mothers and their babies enrolled on Option B+ from health facilities in central region of Uganda. The study was about reducing mother to child transmission of HIV. They found 83.0% of the HIV exposed infants received daily NVP from birth to 6 weeks postpartum [7]. Those that missed nevirapine were estimated at 17%. Sample size estimation was made using this study. The sample size was obtained using the Kish Leslie formula for sample size estimation for proportions [8].

$$\textrm{N}=\textrm{Z}{\alpha}^2\textrm{P}\ \left(1-\textrm{P}\right)/{\textrm{W}}^2$$

N is the sample size required

W is the total width of confidence interval, if precision has been set as 5% then W = 0.05.

Zα is the standard normal value corresponding to the 95% level of confidence (1.96).

P is the estimated proportion of HEI who didn’t receive NVP in a previous study (17%)

$${\displaystyle \begin{array}{l}\textrm{N}={(1.96)}^2\textrm{X}\;0.17\ \textrm{X}\ (0.83)/{(0.05)}^2\\\mathrm{N}=217\ \textrm{participants}\end{array}}$$

Calculated sample size with a 5% non-response rate was 228 participants

#### Sample size for factors associated with missing nevirapine prophylaxis

We used a study by Karcher et al. in which outcomes of different NVP administration strategies in preventing mother-to-child transmission (PMTCT) programs in Tanzania and Uganda were studied. The study revealed that maternal age less than 25 years was significantly associated with the HIV exposed infant missing NVP [9], sample size was calculated using the formula below [10].

$$N=\frac{{\left[{Z}_{\alpha /2}\sqrt{p\left(1-p\right)\left(^{1}\!\left/ \!_{{q}_1}\right.+^{1}\!\left/ \!_{{q}_2}\right.\right)}+{Z}_{\beta}\sqrt{p_1\left(1-{p}_1\right)^{1}\!\left/ \!_{{q}_1}\right.+{p}_2\left(1-{p}_2\right)^{1}\!\left/ \!_{{q}_2}\right.}\right]}^2}{{\left({p}_1-{p}_2\right)}^2}$$

- N is required sample size
- q_1_ is proportion of subjects in group 1, 0.56
- q_2_ is proportion of subjects in group 2, 0.44
- Z_α_ is standard normal value corresponding to 0.05level of significance,1.96
- Z_β_ is standard normal value corresponding to 80% power of the study, 0.84
- p_1_ is proportion in group 1 that have outcome of interest, 0.63
- p_2_ proportion in group 2 that have the outcome of interest,0.74
- p = p_1_q_1_ + p_2_q_2_
- *p* = 0.70
- *N* = 554 participants.

Since the actual sample size of 554 is bigger than the actual number of HIV exposed infants below 6 months of age that are seen at ACU and immunisation clinic in Mulago hospital, we applied the finite population correction factor to arrive at the required sample size.

$$n-\frac{n_0}{1+\frac{\left({n}_0-1\right)}{N}}$$

Where n is the adjusted sample size.

n_0_ is the actual number of HIV exposed infants below 6 months of age.

n_0_ is the unadjusted (n_0_ = 554).

N is the actual number of HIV exposed infants below 6 months of age who are seen at ACU and immunisation clinic. Approximately 280 HIV exposed infants may be seen in a period of 6 months in both ACU and immunisation clinic. Hence, *N* = 280.

Therefore, *n* = 186

We considered the larger sample size of 228 participants

### Sampling procedure

Convenience sampling was done to enroll mother-infant pairs who met the inclusion criteria. This was done from Monday to Friday from both ACU and immunization clinic because there are few HIV exposed infants seen in Mulago hospital. Majority of HIV positive mothers and their HEI are seen in various PMTCT and EID clinics in various health centers within Kampala.

The study team consisted of the principal investigator (PI) and 2 research assistants (RA) who all participated in data collection. Mothers with infants aged 6 months and below who came to the immunization clinic or were admitted at ACU, were asked whether they had done an HIV test in the past 1 month and if not, they were asked to do an HIV test which was done to determine the exposure status of the infant. If found positive, then the infant was declared HIV exposed and the mother-infant pair was enrolled into the study. Mothers who were already HIV positive with adequate documentation of HIV positivity were also enrolled into the study.

Using a structured and pretested study questionnaire of 34 questions under demographics, socio-economic status and antenatal care details, we obtained details from the mothers of the HEI who were interviewed individually by either the PI or research assistant to find out whether the HEI missed nevirapine prophylaxis and factors associated. The questions were asked in either English or the local language, luganda, according to what a mother preferred to use. Recall bias was one of the limitations faced.

Thereafter, a DNA PCR test was conducted on the HEI if he/she had not done any previously. In case the infant’s HIV status was already known, the available documentation was used to document the HIV status of the infant.

A coloured sticker was then placed on the participant file and immunization card so as to avoid enrolling participants more than once into the study.

### Data management and analysis

Codes used as study participant unique ID numbers were assigned and used in chronological order. Study participant unique ID numbers were used for purposes of confidentiality. The PI checked the questionnaire for accuracy, completeness and consistency at the end of each day. All data was entered into the computer using a data entry template of Epidata version 3.1. software package with in-built quality control checks. The final data was stored, backed up and exported to STATA version 14.0 for analysis. The data was analyzed at univariate, bivariate and multivariate levels.

### Univariate analysis

Demographic characteristics were summarized as frequencies and percentages for categorical variables; means and standard deviations or medians and interquatile ranges for continuous variables. These are displayed as tables, and charts. The number of HEI aged 0-6 months that missed nevirapine was expressed as a percentage of the total number of participants recruited.

### Bivariate analysis

Logistic regression models were used to quantify the associations between independent categorical variables (sex, age, religion, education level, household income, undergone PMTCT counselling, ANC visits, HIV tests done during ANC, prematurity, low birth weight, infant hospitalization at birth, mode of delivery, place of delivery, disclosure of HIV status to partner, household income) and the dependent variable (missed nevirapine prophylaxis). The associated *p*-values from the models were used to determine the level of statistical significance. The variables that had a p-value of less than 0.2 and highlighted in literature (undergone PMTCT, disclosure of HIV status, marital status, taking ARVs) were considered in the multivariable model.

### Multivariate analysis

Independent variables that tested significant at bivariate level were be fitted in the binary logistic regression model to generate prevalence ratios and probability values adjusting for several factors (undergone PMTCT, disclosure of HIV status, marital status, taking ARVs) as presented in the results. All *p* values less than 5% were considered significant. We used a forward model building technique to establish the associated factors.

## Results

A total of 240 mother-infant pairs were screened for the study. Seven pairs were excluded because two of the care takers were not the mother and five did not consent. A total of 228 pairs were enrolled.

### Demographic characteristics

One hundred thirty-seven infants were male (57.9%). The mean age of the infants was 4.0 months ±0.3. Two hundred sixteen were born at term (94.7%) and 63 (27.6%) were hospitalized at birth.

Most mothers 127 (55.7%) were aged 26-30. Mothers who were on ART were 209 (91.7%), 126 (55.3%) had disclosed their HIV status to partner were, 75 (32.9%) attended ANC with partner, 191 (83.8%) underwent PMTCT counselling and 184 (80.7%) delivered within a government facility (Table 1).

**Table 1:** Showing demographic maternal characteristics

Variable	HEI who received NVP *n* = 178(%)	HEI who missed NVP *n* = 50(%)	Total *N* = 228
Age in years			
<=19	8(66.7%)	4(33.3%)	12
20-25	49(76.6%)	15(23.4%)	64
26-30	97(76.4%)	30(23.6%)	127
> 30	24(96.0%)	1(4.0%)	25
Taking ARVs			
Yes	173(82.8%)	36(17.2%)	209
Disclosure of HIV status to partner			
Yes	112(88.9%)	14(11.1%)	126
Attended ANC with partner			
Yes	69(92.0%)	6(8.0%)	75
Underwent PMTCT counselling			
Yes	168(88.0%)	23(12.0%)	191
When was the 1st ANC visit			
1st trimester	44(9.6%)	3(6.4%)	47
2nd trimester	107(79.9%)	27(20.2%)	134
3rd trimester/no visit	27(62.8%)	20(37.2%)	43
Place of delivery			
Gov’t facility	157(85.3%)	27(14.7%)	184
Non gov’t facility	21(47.7%)	23(52.3%)	44
Household income			
> 200,000/=($50)	49(68.1%)	23(31.9%)	72
200,000/=($50)- 50,000/=($15)	101(82.8%)	21(17.2%)	122
< 50,000/=($15)	28(82.4%)	6(17.6%)	34

*PMTCT* Prevention of mother to child transmission: *Gov’t* Government: *ARVS* Antiretrovirals: *ANC* Antenatal care

Factors associated with HEI missing NVP prophylaxis at bivariate analysis are shown in Table 2 and at multivariable analysis, delivery from outside government health facilities [AOR = 8.41 95% (CI 3.22-21.99)], mothers not undergoing PMTCT counselling [AOR = 12.01 95% (CI 4.53-31.87)], mothers not on ART [AOR = 8.47 95% (CI 2.06-34.88)] and mothers not having disclosed their HIV status to their partners [AOR = 2.80 95% (CI 1.13-6.95)] were significant factors (Table 3).

**Table 2:** Showing bivariate analysis of maternal factors associated with HEI missing nevirapine prophylaxis

Variable.	Number x/n (50/228)	Percentage (%) (row percentage)	Unadjusted Odds ratio (95% CI)	*p*-value
Age in years				
</= 19	**4/25**	**16.0%**	**1.00**	
20-25	**15/64**	**23.4%**	**0.61(0.16-2.33)**	**0.471**
26-30	**30/127**	**23.6%**	**0.62(0.17-2.20)**	**0.459**
> 30	**1/25**	**4.0%**	**0.08(0.01-0.86)**	**0.037**
Taking ARVs				
Yes	**36/209**	**17.2%**	**1.00**	
No	**14/19**	**73.7%**	**13.46(4.55-39.81)**	**0.001**
Duration of taking ARVs				
< 3 months	**16/23**	**69.6%**	**1.00**	
12 months	**8/47**	**17.0%**	**0.09(0.03-0.29)**	**0.001**
> 1 year	**12/139**	**8.63%**	**0.04(0.01-0.12)**	**0.001**
Disclosure of status to partner				
Yes	**14/126**	**11.1%**	**1.00**	
No	**36/102**	**35.3%**	**4.36(2.19-8.70)**	**0.001**
Attending ANC with partner				
Yes	**6/75**	**8.0%**	**1.00**	
No	**44/153**	**28.8%**	**4.64(1.87-11.50)**	**0.001**
Underwent PMTCT counselling				
Yes	**23/191**	**12.0%**	**1.00**	
No	**27/37**	**73.0%**	**19.72(8.44-46.06)**	**0.001**
First ANC visit				
1st trimester	**3/47**	**6.4%**	**1.00**	
2nd trimester	**27/134**	**20.2%**	**3.70(1.06-12.87)**	**0.040**
3rd trimester and no visit	**20/47**	**42.55%**	**10.86(2.94-40.17)**	**0.001**
Place of delivery				
Gov’t facility	**27/184**	**14.7%**	**1.00**	
Non gov’t facility	**23/44**	**52.3%**	**6.37(3.10-13.09)**	**0.001**

*Gov’t* Government: *ARVS* Anti-retrovirals: *ANC* Antenatal care: *PMTCT* Prevention of mother to child transmission

**Table 3:** showing multivariate analysis of factors associated with missing NVP prophylaxis

Variable	Adjusted Odds ratio (95% CI)	p-value
PMTCT counselling		
Yes	1.00	
No	12.01(4.53-31.87)	0.001
Place of delivery		
Gov’t facility	1.00	
Non gov’t facility	8.41(3.22-21.99)	0.001
Taking ARVs		
Yes	1.00	
No	8.47(2.06-34.88)	0.003
Disclosure of status to partner		
Yes	1.00	
No	2.80(1.13-6.95)	0.026
First ANC visit		
1st trimester	1.00	
2nd trimester	2.84(0.70-11.48)	0.142
3rd trimester and no visit	2.76(0.61-12.42)	0.185

*Gov’t* Government: *PMTCT* Prevention of mother to child transmission: *ARVS* Anti-retrovirals: *ANC* Antenatal care.

## Discussion

One out of five HEI in our study (21.9%) missed nevirapine (NVP) prophylaxis within the first 72 hours. This is high and is explained by the fact that the facility receives patients from various regions of the country. It is therefore a reflection of gaps in provision of NVP prophylaxis at birth in the various health facilities. This is a hindrance to achieving elimination of mother to child transmission (eMTCT). The eMTCT strategy comprises a package of interventions summarized in four approaches; primary prevention of HIV infection, prevention of unintended pregnancies among women living with HIV, prevention of HIV transmission from women living with HIV to their infants and provision of treatment, care, and support to women infected with HIV, their children and their families [11]. Nevirapine prophylaxis for HIV-exposed infants is one of the interventions used in the approach of prevention of HIV transmission from women living with HIV to their infants. In resource-limited settings as such our country, Uganda, daily administration of nevirapine to the infants of mothers infected with human immunodeficiency virus (HIV) can prevent breast-milk HIV transmission [12]. Babies not receiving nevirapine is associated with increased risk of mother to child transmission of HIV [7]. However, our study had a limitation of not stratifying exactly where these babies were born and first given the NVP prophylaxis. We therefore cannot conclude on which specific facilities experience the gap of not receiving NVP within the first 72 hours of life.

A retrospective study done in 2013 in 145 health facilities in 24 districts of central Uganda, by Muhumuza et al. found that 17.0% of the HIV exposed infants did not receive daily NVP from birth to 6 weeks postpartum [7]. This study was done during the early phase of Option B+ roll-out in Uganda and could explain the slightly lower percentage of missed NVP compared to our study which found 21.9% of the HIV exposed infants not having receive NVP prophylaxis within the first 72 hours of life. A possible explanation of this could be because compliance to eMTCT guidelines in 2013 was found to be high. This however still shows a great hindrance to eliminating mother to child transmission of HIV.

Another retrospective study done in northern Uganda revealed 86.9% HEI received NVP prophylaxis from birth until 6-weeks leaving 13.1% who missed NVP prophylaxis [13]. This study aimed to find out retention of HEI and associated factors. The percentage could have been lower than our findings of 21.9% because they conducted a retrospective study and acknowledged the fact that they used secondary data which was initially collected for clinical care rather than research purposes. Consequently, the numbers of variables were limited and the exclusion of HEIs with missing data also reduced the sample size that otherwise would have improved the precision of the results. Our study being a cross-sectional study was able to utilize as much data as possible to estimate the number of HEI that missed NVP prophylaxis.

In a cross-sectional surveillance study of mother-infant pairs using umbilical cord blood samples collected between June 10, 2007, and October 30, 2008, from 43 randomly selected facilities providing delivery services in Cameroon, Côte d’Ivoire, South Africa, and Zambia, there was random sampling of sites with services to prevent mother-to-child HIV transmission done. A percentage of 49% of HIV-exposed infants received the minimal regimen of single-dose nevirapine [14]. Continued missed NVP prophylaxis reflected in these studies hinders eMTCT. Our study shows that even with continued strides towards elimination of vertical transmission, we still have to address some bottlenecks like missing NVP prophylaxis within the first 72 hours of life that is a hindrance to this cause.

### Factors associated with missing nevirapine prophylaxis

Mothers who had not undergone PMTCT counselling were 12 times more likely to have HEI missing nevirapine prophylaxis. New drug regimens and more comprehensive treatment guidelines for PMTCT have helped mitigate pediatric infection rates, especially in high-burden, low-resource settings [3, 15]. Karcher et al. found that mothers who had not undergone PMTCT counselling at a hospital were 65.5% likely to miss infant NVP prophylaxis [9]. Both maternal ART and infant NVP prophylaxis strategies were found to be safe and associated with very low breastfeeding HIV transmission and high infant HIV free survival at 24 months [4]. Our study demonstrated that mothers who had not undergone PMTCT counselling were more likely to have HEI missing NVP prophylaxis hence further mother to child HIV transmission.

Mothers who delivered from outside a health facility were 8.4 times more likely to miss nevirapine prophylaxis and hence increased risk of HIV transmission. Delivery within a government health facility increases the chances of an infant receiving nevirapine prophylaxis hence reducing on the possibility of missing NVP [5]. The Private Health Support (PHS) Program strengthened systems to eliminate mother-to-child HIV transmission and as a result, there was a reduction from 5 to < 2% [16]. Therefore, a number of non-governmental health facilities have been empowered to eliminate mother to child HIV transmission. However, delivery outside a health facility such as at a Traditional Birth Attendant (TBA) increased the risk of missing NVP prophylaxis as observed in our study.

Mothers who were not on ART were 8.5 times more likely to have an HEI that missed nevirapine. The risk of mother-to-child transmission of HIV is higher among women who are not on antiretroviral therapy [17]. It is common for women to gradually stop taking ARV drugs after giving birth, which not only compromises their health but also puts their infant’s at an increased risk of acquiring HIV during breastfeeding [18].In a study done in Cameroon, HIV transmission rate differed by maternal prophylaxis: 1.7% for HAART, 2.7% for dual therapy and 15.7% for mothers who were not receiving ART with a *p*-value of < 0.001 [19]. A study carried out in central Uganda found that among mothers taking ARVs, the levels of maternal adherence to NVP prophylaxis regimens were above 70% [6]. This explains the correlation between taking ART and infant nevirapine syrup uptake. Our study showed that mothers who were not on ART had higher chances of having HEI missing NVP prophylaxis hence increasing the chances of vertical HIV transmission.

### HIV infection among HEI who missed NVP

Almost three quarters of HEI who missed NVP prophylaxis were HIV infected. In this study, there was a 70% risk of HIV positivity among HEI that missed NVP prophylaxis. With the proven efficacy of nevirapine, HIV infection is lower in infants who take nevirapine compared to those who do not. The SWEN study showed that once-daily infant nevirapine for 6 weeks resulted in a 53% reduction in postnatal HIV infection at age 6 weeks [20].A study done by Kahungu et al. found that HEI who did not receive ART prophylaxis at birth were five times more likely to be HIV infected than those who received prophylaxis [21].

This study also found that 5 out of 40(12.5%) HEI were HIV infected despite receiving nevirapine prophylaxis. This is in sync with the fact that undetectable viral load in a mother and infant prophylaxis does not equate to non-transmission of HIV virus to the infant [18].

Our study had various limitations as stated below. We were unable to stratify the exact health facilities where the HEI were born. We were however, able to classify them into government facilities and non-government facilities. We were unable to ascertain whether a mother sero-converted in the postnatal period or not in cases where mothers were found positive postnatally so we assumed mothers had been positive even during pregnancy. We were unable to confirm that an HEI had vertical transmission of HIV. However, since these were infants aged less than 6 months, the commonest mode of transmission of HIV is vertical transmission. The study tackled the issue of recall bias by confirming given information with available documents.

## Conclusion

One in five HEI in this study missed their nevirapine prophylaxis. Factors significantly associated with infants missing nevirapine prophylaxis were; mothers not undergoing PMTCT counselling, mothers not on ARVs, non- disclosure of HIV status to their partners and delivering from outside a government health facility. Nearly three quarters of HEI who missed NVP were HIV infected. Mothers should be encouraged to deliver within health facilities. There should be more emphasis on infant NVP prophylaxis through interventions that strengthen PMTCT counselling, and accreditation of more private facilities to offer PMTCT services. We recommend a bigger study carried out in various health facilities in the country, both government and non government to give a clear picture on uptake of NVP prophylaxis in the various health facilities.

## Data Availability

The datasets used and/or analysed during the current study are available from the corresponding author on reasonable request.

## Abbreviations

ART: Anti-retroviral therapy
HEI: HIV exposed infant
KII: Key Informant Interview
MoH: Ministry of Health
MNRH: Mulago National Referral Hospital
MTCT: Mother to Child Transmission
NVP: Nevirapine
PCR: Polymerase Chain Reaction
PMTCT: Prevention of Mother to Child Transmission
